# Efficacy of *Mentha aquatica* L. Essential Oil (Linalool/Linalool Acetate Chemotype) against Insect Vectors and Agricultural Pests

**DOI:** 10.3390/ph16040633

**Published:** 2023-04-21

**Authors:** Marta Ferrati, Eleonora Spinozzi, Cecilia Baldassarri, Filippo Maggi, Roman Pavela, Angelo Canale, Riccardo Petrelli, Loredana Cappellacci

**Affiliations:** 1 Chemistry Interdisciplinary Project (ChIP), School of Pharmacy, University of Camerino, Via Madonna delle Carceri, 62032 Camerino, Italy; 2 Crop Research Institute, Drnovska 507, 161 06 Prague, Czech Republic; 3 Department of Plant Protection, Czech University of Life Sciences Prague, Kamycka 129, 165 00 Prague, Czech Republic; 4 Department of Agriculture, Food and Environment, University of Pisa, Via del Borghetto 80, 56124 Pisa, Italy

**Keywords:** bio-insecticide, bio-pesticide, *Culex quinquefasciatus*, *Metopolophium dirhodum*, *Spodoptera littoralis*, *Tetranychus urticae*, *Musca domestica*

## Abstract

In recent years, agrochemical industries have been focused on the development of essential oil (EO)-based biopesticides, which can be considered valuable alternatives to traditional chemical products. The genus *Mentha* (Lamiaceae) comprises 30 species characterized by a wide range of biological activities, and some of their EOs showed good potential as pesticidal agents. In this regard, the aim of this study was to evaluate the insecticidal activity of the EO obtained from a rare linalool/linalool acetate chemotype of *Mentha aquatica* L. The EO was found to be highly effective against *Culex quinquefasciatus* (Say) 2nd instar larvae, *Metopolophium dirhodum* (Walker) adults, *Spodoptera littoralis* (Boisduval) 2nd instar larvae, and *Tetranychus urticae* (Koch) adults, showing lethal concentrations (LC_50_) or doses (LD_50_) of 31.5 ± 2.2 µL L^−1^, 4.9 ± 0.8 mL L^−1^, 18.5 ± 2.1 µg larvae^−1^, and 3.3 ± 0.5 mL L^−1^, respectively. On the contrary, *Musca domestica* L. adults and 3rd instar larvae of *C. quinquefasciatus* and *S. littoralis* were moderately affected by the treatment (LC_50_ or LD_50_: 71.4 ± 7.2 µg adult^−1^, 79.4 ± 5.2 µL L^−1^, 44.2 ± 5.8 µg larvae^−1^, respectively). The results obtained in this work demonstrated that various insects and pests could be differently sensible to the same EO and may lead to the exploitation of this plant or its major volatile compounds as novel ingredients of botanical insecticides and pesticides.

## 1. Introduction

The genus *Mentha* belongs to the Lamiaceae family and comprises approximately 30 species distributed all over the world [1]. Plants of this genus have been widely used for several purposes by the pharmaceutical, nutraceutical, food, beverage, and tobacco industries [2]. They represent the most exploited sources for the extraction of essential oils (EOs), which are produced at a rate of 23,000 metric tons every year for a value of $400 million [3].

In addition, several species of this genus have been proven as good sources for botanical insecticide ingredients due to their capacity to produce EOs equipped with contact toxicity, fumigant, and repellent effects against a wide spectrum of target insects, such as storage pests, vectors, and larvae [4]. The most investigated species of this genus are *Mentha* x *piperita* L., *Mentha spicata* L., and *Mentha pulegium* L.

*Mentha aquatica* L., also known as ‘water mint’, is a member of this genus growing in wet environments of Europe, North Africa, and West Asia. Moreover, it has been recently introduced into America and Australia [5,6]. The ethnobotanical uses reported for this plant have been mainly associated with its medicinal value, as *M. aquatica* is currently employed as a remedy for colds, respiratory, and gastrointestinal problems. Particularly, the gastrointestinal effect depends on the modulation of non-protein sulfhydryl substances, nitric oxide, and gastric secretion [7]. In addition, the leaves of the plant are smoked in South Africa to treat mental diseases [8], and the central nervous system activity has been associated with a strong affinity to the GABA-benzodiazepine receptor [6]. *Mentha aquatica* also showed butyrylcholinesterase inhibitory activity and antioxidant, antimicrobial, catalytic, and cytoprotective actions [2,9,10]. However, most of the available studies on *M. aquatica* mainly focus on the chemical variability of its EO, which is in turn related to its geographic origins and to the agronomic treatments applied when it is cultivated as a crop. Currently, the reported chemotypes of *M. aquatica* are dominated by menthofuran, pulegone, menthol, piperitone oxide, or linalool [3,11,12,13,14,15]. To the best of our knowledge, the *M. aquatica* EO has been poorly explored for potential insecticidal effects when compared with other representatives of the genus *Mentha*.

In recent decades, the exploitation of botanical products capable of replacing traditional chemical pesticides has exponentially increased [16,17,18,19]. Indeed, problems related to food safety and environmental pollution have led to greater attention to sustainability, also in the agrochemical sector [20,21]. Although pesticides are essential for crop protection and, consequently, for food production, chemical residues can be toxic to other non-target organisms and have a negative impact on various environmental media such as air, soil, and water [22]. Therefore, replacing chemical substances with botanical products results in a good compromise to guarantee the protection of crops without causing damage to the environment, humans, or non-target species [23,24]. Among botanical products, EOs could be potential candidates for the development of novel biopesticides and insecticides. In previous studies, we showed that numerous EOs could display their insecticidal and pesticidal potential towards different insect species depending on the synergistic or antagonistic effect of their components that revealed suitable LC_50_ and LC_90_ values [25,26,27].

In this context, given the interest in *Mentha* species as sources of natural insecticides and pesticides against different vectors and stored grain pests [4,28,29], we evaluated for the first time the insecticidal and acaricidal potential of a linalool /linalool acetate-rich EO of *M. aquatica*. In order to provide evidence of the wide spectrum of insecticidal efficacy of this EO, we selected insect vectors transmitting diseases to humans and arthropods spreading on several crops, causing significant economic losses globally. In detail, the *M. aquatica* EO was tested on two species of public health relevance, i.e., *Culex quinquefasciatus* (Say) (Diptera: Culicidae) and *Musca domestica* (L.) (Diptera: Muscidae), and three representatives of important agricultural pests— *Metopolophium dirhodum* (Walker) (Hemiptera: Aphididae), *Spodoptera littoralis* (Boisduval) (Lepidoptera: Noctuidae), and *Tetranychus urticae* (Koch) (Acari: Tetranychidae).

## 2. Results

### 2.1. EO Chemical Composition

Table 1 shows the results derived from gas chromatography-mass spectrometry (GC-MS) analysis of *M. aquatica* EO, which was dominated by the presence of oxygenated monoterpenes (86.9%), accompanied by minor percentages of monoterpene hydrocarbons (6.2%), sesquiterpene hydrocarbons (3.2%), esters (1.0%), and oxygenated sesquiterpenes (0.7%), which together accounted for 98.1% of the total composition. The main representatives of the oxygenated monoterpenes, as well as main EO constituents, were linalool acetate (34.9%) and linalool (26.8%), while minor components were *α*-terpinyl acetate (12.3%), 1,8-cineole (6.7%), and *α*-terpineol (5.1%). Among monoterpene hydrocarbons, myrcene (2.0%), limonene (0.9%), and (*E*)-*β*-ocimene (0.9%) were the most abundant, while germacrene D (1.8%) and (*E*)-caryophyllene (1.3%) were the most representative compounds of the sesquiterpene hydrocarbons.

### 2.2. Insecticidal and Acaricidal Efficacy

Regarding the insecticidal and acaricidal efficacy of *M. aquatica* EO, it was found to cause relatively good acute toxicity to all the target species. The estimated lethal doses (LD_50_) or concentrations (LC_50_) are shown in Table 2. The EO was more effective on younger larval instars. For instance, the LC_50_ for *C. quinquefasciatus* was estimated at 31.5 µL L^−1^ for the 2nd instar and at 79.4 µL L^−1^ for the 3rd larval instar. The same trend was found for 2nd and 3rd instar larvae of *S. littoralis* (LD_50_ = 18.5 and 44.2 µg larva^−1^, respectively). Very good effectiveness of the EO was found for small pests, such as the adults of *M. dirhodum* and *T. urticae* tested by us, for which the LC_50_ was estimated at 4.9 and 3.3 mL L^−1^, respectively. Conversely, low efficacy was found for *M. domestica* adults (LD_50_ = 71.4 and 50.5 µg adult^−1^; LD_90_ = 329.8 and 462.6 µg adult^−1^, for females and males, respectively).

## 3. Discussion

It is well known that the chemical composition of EOs is linked to several endogenous and exogenous factors, such as chemotypes, geographical distribution, growing conditions and climate, time of collection, and extracting techniques [31]. This chemical variability has also been reported for *M. aquatica* EO, for which the main varieties described in the literature are reported in Table 3. The composition found in our study is similar to that of other cultivated populations of *M. aquatica*, being linalool and linalool acetate the main compounds, even if at different ratios. For instance, for plants cultivated in Iran, the EO was mainly characterized by the presence of linalool (37.8%) and linalool acetate (30.6%) [32], as well as for species collected in India, for which the amount of these two compounds varied according to the season of collection. In fact, linalool was the dominant compound for plants collected from April to September (25.2–48.4%), while linalool acetate was the dominant compound for those collected from October to December (42.1–48.0%). A similar chemical constitution was also found for *M. aquatica* var. *citrata,* for which linalool and linalool acetate were the most representative compounds [14].

On the other hand, the chemical composition herein described contrasts with those reported from other studies. In fact, menthofuran has sometimes been reported as the most abundant compound. This is the case for the EO obtained from wild-growing plants in Vojvodina (16.9%), as well as the ones obtained from wild populations in Ethiopia (70.5%) and Romania (51.3–58.6%) [3,5,36]. The predominance of menthofuran in the EO seems to be also linked to other growing conditions, as in the case of *M. aquatica* plants growing in presence of *Chrysolina herbacea* (Duftschmid 1825) (Coleoptera: Chrysomelidae). In these conditions, the plant activates some genes involved in the biosynthesis of terpenoids and redirects them to the production of menthofuran, which was demonstrated to repel *C. herbacea* [37]. The preponderance of menthofuran was also correlated with genetic factors [38,39].

The genus *Mentha* has been extensively studied for its insecticidal and acaricidal activity against agricultural pests and insect vectors, and some species have shown great efficacy [28,29]. For example, *Mentha longifolia* (L.) and *Mentha suaveolens* (Ehrh.) have demonstrated high larvicidal activity against third instar larvae of *C. quinquefasciatus* after 24 h of exposure with LC_50_ values of 17 mg L^−1^ for both EOs, which were characterized by the main compound piperitone oxide [40]. In a study, among 34 EOs from different *Mentha* species, *M. pulegium* was found to be the most effective against *M. domestica* adults under laboratory conditions in fumigant and topical bioassays, with LD_50_ values of 13 μg fly^−1^ and 4.7 μg cm^−1^, respectively [41]. Its EO was dominated by pulegone, the main responsible for the biological activity. Moreover, *M. piperita* caused >90% mortality, while *M. spicata* caused 81–82% mortality at 14 × 10^−3^ μL mL^−1^, demonstrating a significant acaricidal effect against *T. urticae*; in this case, menthol and carvone usually represent the main compounds of the EOs for the two species, respectively [42].

Despite the large body of relevant literature regarding the potential of the *Mentha* species to be used for the control of several vectors and pests, studies concerning the linalool/linalool acetate chemotype’s insecticidal activity have not yet been reported. This is the first study recording useful information for the potential development of biopesticides exploiting the rare chemotype of this species from Lebanon. However, both linalool and linalool acetate have been revealed to be effective pesticides in several studies [4,43,44,45,46]. Linalool has been demonstrated to be a competitive acetylcholinesterase inhibitor [46,47], and both linalool and linalool acetate seem to interfere with the insect central nervous system, in particular interacting with glutamatergic transmission and the GABA_A_ receptor [48,49,50]. Indeed, EOs containing linalool and/or linalool acetate have been reported as effective insecticidal agents. For instance, basil EO showed a promising insecticidal potential on targets such as *Rhyzopertha dominica* L. (75.0% mortality at 4% of EO) [51], *Sitophilus oryzae* L. (LC_50_ of 4.9 μL mL^−1^) [52], *Ceratitis capitata* Wiedemann (LT_90_ of 17.0 min), *Bactrocera dorsalis* (Hendel) (LT_90_ of 26.0 min), and *B. cucurbitae* Coquillett (LT_90_ of 32.0 min) [53]. This effect has been mainly linked to the high levels of linalool in the EO. In the same way, the EO from *Cinnamomum camphora* Ness and Eberm var. *linaloolifera* Fujita, which is characterized by linalool as the main compound, has been reported for its insecticidal properties against *Anticarsia gemmatalis* Hübner (LC_50_ of 0.908% *v*/*v*) [54] and *Trialeurodes vaporariorum* Westwood (nymph mortality of 88.5% at 2.0% *v*/*v*) [55]. Similarly, *Coriandrum sativum* L. seeds’ EO displayed an insecticidal potential on adults of *Tribolium confusum* Duval (LC_50_ of 1.34 μL L^−1^ air) and *Callosobruchus maculatus* Fabricius (LC_50_ of 318.02 μL L^−1^ air), and this action was correlated to the predominant presence of linalool [56]. On the other hand, the EO from *Myrtus communis* L., mainly characterized by linalool and linalool acetate, displayed insecticidal action on three stored-product insects, namely *Ephestia kuehniella* Zeller (LC_50_ of 12.7 μL L^−1^ air), *Plodia interpunctella* Hübner (LC_50_ of 22.6 μL L^−1^ air), and *Acanthoscelides obtectus* Say (LC_50_ of 49.6 μL L^−1^ air) [57]. In addition, *Cananga odorata* (Lam.) Hook. f. and Thomson EO showed marked contact toxicity against *Sitophilus zeamais* Motschulsky with an LD_50_ value of 33.1 µg adult^−1^ and fumigant toxicity with an LC_50_ value of 14.8 mg L^−1^ [58]. In our work, we did not have a positive control available; however, we can compare the effectiveness of EO with the positive control of previously published works in which the same insect species were used in the same developmental stages and the application was carried out in a similar way with the same genetic material of the target organisms and under similar post-application conditions. Regarding the herein presented study, *M. aquatica* EO was found to be more effective than Rock Effect (a commercial biopesticide based on *Pongamia pinnata* L. oil), which was used as a positive control by Pavela et al. [59] and tested against the same targets. Specifically, the LD_50_ or LC_50_ values were higher for the positive control (˃500 μg adult^−1^, 275.4 μg mL^−1^, 12.5 mL L^−1^, 5.8 mL L^−1^, 3.3 ± 0.5 mL L^−1^, respectively) than for the *M. aquatica* EO (71.4 ± 7.2 μg adult^−1^, 79.4 ± 5.2 μg mL^−1^, 4.9 ± 0.8 mL L^−1^, respectively) when both were tested against *M. domestica* female adults, *C. quinquefasciatus* 3rd instar larvae, and *M. dirhodum* adults. On the other hand, their activity is quite comparable in the test against *S. littoralis* (LD_50_ of 18.2 and 18.5 µg larva^−1^ for the positive control and *M. aquatica* EO, respectively). The effectiveness of *M. aquatica* EO was of varying degrees of intensity, as the different species of insects tested were differently sensitive to the same EO.

## 4. Materials and Methods

### 4.1. Plant Material and EO Extraction

Leaves of cultivated *M. aquatica* were manually collected in Kafarkela (33°17’ N 35°33’ E, 400 m a.s.l.), Southern Lebanon, in August 2019. The botanical identification was performed by Dr. Fabrizio Bartolucci, University of Camerino, Floristic Research Center of the Apennines. A voucher specimen was stored in the herbarium of the Floristic Research Centre of the Apennines under the voucher codex APP No. 66212. *Mentha aquatica* EO was obtained by hydrodistillation of dried leaves using a Clevenger-type apparatus for 4 h. The calculation of the oil yields was based on a dry weight (*w*/*w*) basis and resulted in 3.35%.

### 4.2. GC–MS Analysis of Essential Oils

The GC–MS analysis was carried out with an Agilent 6890N–5973N GC–MS system (Santa Clara, CA, USA) on a sample of *M. aquatica* EO prepared by dilution to 1:100 with *n*-hexane. The instrument was operating in the EI mode at 70 eV and using a HP-5MS (5% phenylmethylpolysiloxane, 30 m, 0.25 mm i.d., film thickness 0.1 µm) (J&W Scientific, Folsom, CA, USA) capillary column.

The chromatographic parameters and chromatogram analysis were the same as those reported by Nkuimi Wandjou et al. [27]. Briefly, the analytical standards of *α*-pinene, sabinene, *β*-pinene, myrcene, *α*-terpinene, *p*-cymene, limonene, 1,8-cineole, (*Z*)-*β*-ocimene, (*E*)-*β*-ocimene, *γ*-terpinene, terpinolene, terpinene-4-ol, *α*-terpineol, (*E*)-caryophyllene, and *α*-humulene were purchased from Merck (Milan, Italy) and used for peak assignments based on retention time and mass spectrum (MS). Moreover, the combination of the calculated linear retention index (RI) and MS was used to confirm the identity of the other compounds. Semi-quantitative values (peak area percentages) were obtained by peak normalization without using correction factors.

### 4.3. Target Insects and Mites

As target arthropod species, we tested *C. quinquefasciatus*, *M. domestica*, *M. dirhodum*, *S. littoralis*, and *T. urticae*. These species have been reared under controlled laboratory conditions at the Crop Research Institute (Prague, Czech Republic) for more than 20 generations.

Arthropod mass rearing in brief: *C. quinquefasciatus* larvae were fed with dry dog biscuits; adults were allowed to mate; females were fed with blood in order to complete their egg development. Eggs were laid in unprepared containers of water. *M. domestica* larvae were fed a diet developed at the Crop Research Institute (Prague, Czech Republic), which was composed of sawdust, milk, and agar. Housefly adults were fed sugar solutions and powdered milk. Eggs were laid on cotton wool dipped in sweet milk. Wheat plants in pots with ordinary substrate were selected to rear *M. dirhodum*. *S. littoralis* larvae were fed with agar, soybean meal, and vitamins; adults, fed with honey solution, mated and laid eggs on filter paper previously prepared. Bean plants grown in a common garden substrate were selected to rear *T. urticae* in a growth chamber. All arthropod target species were maintained at 25 ± 1 °C, 70 ± 3% R.H., and 16:8 h (L:D). Experiments described thereafter were carried out under the same conditions [59].

### 4.4. Insecticidal and Acaricidal Activity

The *M. aquatica* EO was diluted in acetone (p.a., Sigma Aldrich, Prague, Czech Republic) to obtain various concentrations (applied at 1 μL): for *S. littoralis* larvae, 10, 20, 30, 40, 50, 60, 70, 80, 90, and 100 μg larva^−1^; and for *M. domestica* adults, 50, 80, 100, 150, 200, 250, and 300 μg adult^−1^. Before application, the arthropods were anesthetized with CO_2_. Acetone was the negative control. After treatment, the tested organisms were placed into the rearing containers (15 × 12 × 8 cm) equipped with a perforated lid and fed with the aforementioned diet. The experiments were replicated four times; each replicate was performed with 20 individuals. For *C. quinquefasciatus* larvae, EO was dissolved in DMSO (dimethyl sulfoxide, Merck, Prague, Czech Republic) and tested according to the WHO (1996) procedure [60] with minor modifications. Each time, 1 mL of DMSO, which contained a defined amount of EO, was thoroughly mixed in 99 mL of chlorine-free standing water. In this way, a concentration series containing 20, 40, 60, 80, and 100 mg mL^−1^ of mint EO was obtained. DMSO was used as a negative control. For each replicate, 20 larvae were used, and the experiment was repeated four times. For experiments with *M. dirhodum* and *T. urticae*, first, the EO was emulsified using Tween 80 (Sigma-Aldrich, Prague, Czech Republic) in a 1:1 (*v*:*v*) ratio. Afterwards, different concentrations were prepared (for *M. dirhodum* adults, 2.0, 3.0, 4.0, 5.0, 6.0, and 7.0 mL L^−1^, and for *T. urticae* adults, 1.0, 2.0, 3.0, 4.0, 5.0, and 6.0 mL L^−1^) by thoroughly mixing the modified EO in water. Always, 20 adults of *M. dirhodum* or *T. urticae* (for each replication) were transferred to wheat or bean leaves, respectively, using a fine brush. The plants were located in a flowerpot with a diameter of 9 cm. An electric applicator was used to spray the plants (5 mL of solution per plant) in five replicates.

All experiments were conducted in an air-conditioned room at a temperature of 25 °C, a photoperiod of 16 h of light, and 70–80% relative humidity. Twenty-four hours after the application, the number of dead individuals was determined. All individuals that did not show any movement in response to a mechanical stimulus were considered dead.

For the calculation of lethal doses or concentrations, at least five concentrations or doses for which mortality was found to be in the range of 20–90% were always selected. After correction of mortality by Abbott [61], LD(LC)_50(90)_ were estimated using Probit analysis [62].

## 5. Conclusions

In this work, a linalool acetate/linalool chemotype of *M. aquatica* was tested on *C. quinquefasciatus*, *M. domestica*, *M. dirhodum*, *S. littoralis*, and *T. urticae,* showing a relatively good acute toxicity on most of the tested targets. In detail, for *C. quinquefasciatus* and *S. littoralis,* a higher efficacy of the EO was found on the lower larval stages, while moderate activity was detected on *M. dirhodum* and *T. urticae*. Conversely, the EO was less effective on *M. domestica* adults. The different results obtained in the reported study suggest that various mechanisms of action, likely ascribable to the EO main constituents linalool and linalool acetate, could be involved in the different targets effects, and more studies should be performed to deepen this aspect.

Even though the genus *Mentha* has been widely reported for its insecticidal and acaricidal potential, this is the first study evaluating the above-mentioned properties of *M. aquatica* EO, namely the linalool acetate/linalool chemotype from Lebanon. The results herein presented could represent the starting point for a further exploration of this plant EO and/or its two major constituents as a botanical insecticide and pesticide ingredient.

## Figures and Tables

**Table 1:** Chemical composition of *Mentha aquatica* essential oil.

No	Component ^a^	RI ^b^	RI Lit ^c^	Area % ^d^
1	*α*-thujene	921	924	Tr
2	*α*-pinene	925	932	0.2 ± 0.0
3	sabinene	966	969	0.5 ± 0.1
4	*β*-pinene	968	974	0.6 ± 0.1
5	myrcene	989	988	2.0 ± 0.3
6	*α*-terpinene	1013	1014	0.1 ± 0.0
7	*ρ*-cymene	1022	1020	Tr ^e^
8	limonene	1024	1024	0.9 ± 0.2
9	1,8-cineole	1025	1026	6.7 ± 1.0
10	(*Z*)-*β*-ocimene	1036	1032	0.7 ± 0.1
11	(*E*)-*β*-ocimene	1046	1044	0.9 ± 0.2
12	*γ*-terpinene	1054	1054	0.2 ± 0.0
13	*cis*-sabinene hydrate	1063	1065	Tr
14	terpinolene	1084	1086	0.2 ± 0.0
15	linalool	1100	1095	26.8 ± 2.5
16	isopentyl 2-methyl butanoate	1104	1100	0.4 ± 0.1
17	2-methyl butyl isovalerate	1109	1103	0.1 ± 0.0
18	1-octen-3-yl acetate	1114	1110	0.1 ± 0.0
19	3-octanol acetate	1126	1120	0.3 ± 0.1
20	*δ*-terpineol	1163	1162	Tr
21	terpinen-4-ol	1172	1174	0.2 ± 0.0
22	*α*-terpineol	1185	1186	5.1 ± 0.9
23	nerol	1227	1227	0.2 ± 0.0
24	linalool acetate	1256	1254	34.9 ± 3.1
25	*α*-terpinyl acetate	1345	1346	12.3 ± 1.9
26	neryl acetate	1365	1359	0.7 ± 0.1
27	*β*-bourbonene	1374	1387	Tr
28	(*E*)-caryophyllene	1412	1417	1.3 ± 0.3
29	*α*-humulene	1446	1452	Tr
30	(*E*)-*β*-farnesene	1455	1454	Tr
31	germacrene D	1470	1484	1.8 ± 0.3
32	hedycaryol	1542	1546	0.7 ± 0.1
	Total identified (%)			98.1 ± 0.5
	Grouped compounds (%)			
	Monoterpene hydrocarbons			6.2 ± 0.3
	Oxygenated monoterpenes			86.9 ± 0.7
	Sesquiterpene hydrocarbons			3.2 ± 0.2
	Oxygenated sesquiterpenes			0.7 ± 0.1
	Esters			1.0 ± 0.1

^a^ Components were eluted from a HP-5MS column (30 m l. × 0.25 mm i.d., 0.1 μm f.t.). ^b^ Linear retention index experimentally determined with respect to a mixture of C_7_-C_30_ *n*-alkanes (Sigma-Aldrich) according to Van den Dool and Kratz formula (1963) [30]. ^c^ Retention index value taken from ADAMS or FFNSC3 libraries. ^d^ Peak area relative percentages are the means of two independent injections ± SD. ^e^ Traces, % < 0.1.

**Table 2:** Insecticidal and acaricidal activity of *Mentha aquatica* essential oil against target arthropod pests and vectors.

Target Insect Species	Unit	LD_50_/LC_50_	CI_95_ ^a^	LD_90_/LC_90_	CI_95_ ^a^	Chi	*p*-Level	Df
*Musca domestica*—adults female *Musca domestica*—adults male	µg adult^−1^	71.4 ± 7.2	58.2–85.9	329.8 ± 15.5	298.5–522.7	3.678	0.321	4
	µg adult^−1^	50.5 ± 5.9	48.2–62.8	462.6 ± 25.7	398.8–552.1	3.781	0.203	5
*Culex quinquefasciatus* 2nd instar larvae	µl L^−1^	31.5 ± 2.2	22.8–36.7	80.9 ± 6.7	72.8–91.5	1.512	0.896	4
*Culex quinquefasciatus* 3rd instar larvae	µl L^−1^	79.4 ± 5.2	62.5–98.7	307.2 ± 26.4	285.7–332.5	3.219	0.124	4
*Spodoptera littoralis* 2nd instar larvae	µg larva^−1^	18.5 ± 2.1	15.2–22.9	41.9 ± 2.9	33.8–47.7	0.845	0.985	4
*Spodoptera littoralis* 3rd instar larvae	µg larva^−1^	44.2 ± 5.8	36.9–53.2	117.8 ± 5.1	98.7–123.8	1.169	0.760	3
*Metopolophium dirhodum* adult	mL L^−1^	4.9 ± 0.8	4.5–5.2	7.1 ± 0.3	6.5–8.9	0.891	0.598	3
*Tetranychus urticae* adults	mL L^−1^	3.3 ± 0.5	2.9–3.9	6.2 ± 0.8	5.7–7.3	1.258	0.722	3

^a^ 95% confidence interval relative to LD_50(90)_ LC_50/90_ values.

**Table 3:** Main *Mentha aquatica* essential oil chemotypes.

No	Origin	Major Compound	Reference
**1**	South of Tunisia, Region of Sfax	Pulegone	[11]
**2**	Vojvodina, Serbia	Menthofuran	[33]
	Submediterranean region of south Croatia		[34]
	Ethiopia		[3]
	Pisa, Italy		[35]
	South-east Romania		[5]
**3**	North of Iran, Mazandaran province	Piperitenone oxide	[13]
**4**	West of Iran, Kermanshah province	Menthol	[14]
**5**	Israel	Linalool	[14]
	Western Iran, Lorestan region		[32]

## Data Availability

The data are contained within the article.

## References

[1] Deschamps C., Zanatta J.L., Bizzo H.R., Oliveira M.D.C., Roswalka L.C. (2008). Seasonal evaluation of essential oil yield of mint species. Ciênc. Agrotecnol..

[2] Ferhat M., Erol E., Beladjila K.A., Çetintaş Y., Duru M.E., Öztürk M., Kabouche A., Kabouche Z. (2017). Antioxidant, anticholinesterase and antibacterial activities of *Stachys guyoniana* and *Mentha aquatica*. Pharm. Biol..

[3] Gethaun Z., Asres K., Mazumder A., Bucar F. (2008). Essential oil composition, antibacterial and antioxidant activities of *Mentha aquatica* growing in Ethiopia. Ethiop. Pharm. J..

[4] Kumar P., Mishra S., Malik A., Satya S. (2011). Insecticidal properties of Mentha species: A review. Ind. Crops Prod..

[5] Andro A.R., Boz I., Zamfirache M.M., Burzo I. (2013). Chemical composition of essential oils from *Mentha aquatica* L. at different moments of the ontogenetic cycle. J. Med. Plant Res..

[6] Jäger A.K., Almqvist J.P., Vangsøe S.A.K., Stafford G.I., Adsersen A., van Staden J. (2007). Compounds from *Mentha aquatica* with affinity to the GABA-benzodiazepine receptor. S. Afr. J. Bot..

[7] De Oliveira Braga L.E., da Silva G.G., de Oliveira Sousa I.M., de Oliveira E.C.S., Jorge M.P., Monteiro K.M., Sedano T.C., Foglio M.A., Ruiz A.L.T.G. (2022). Gastrointestinal effects of *Mentha aquatica* L. essential oil. Inflammopharmacology.

[8] Olsen H.T., Stafford G.I., van Staden J., Christensen S.B., Jäger A.K. (2008). Isolation of the MAO-inhibitor naringenin from *Mentha aquatica* L. J. Ethnopharmacol..

[9] Nouri A., Yaraki M.T., Lajevardi A., Rezaei Z., Ghorbanpour M., Tanzifi M. (2020). Ultrasonic-assisted green synthesis of silver nanoparticles using *Mentha aquatica* leaf extract for enhanced antibacterial properties and catalytic activity. Colloid Interface Sci. Commun..

[10] Pereira O.R., Macias R.I., Domingues M.R., Marin J.J., Cardoso S.M. (2019). Hepatoprotection of *Mentha aquatica* L., *Lavandula dentata* L. and *Leonurus cardiaca* L. Antioxidants.

[11] Dhifi W., Litaiem M., Jelali N., Hamdi N., Mnif W. (2011). Identification of a new chemotye of the plant *Mentha aquatica* grown in Tunisia: Chemical composition, antioxidant and biological activities of its essential oil. J. Essent. Oil Bear. Plants.

[12] Djamila B., Zohra K.F., Lahcene K., Zohra R.F. (2021). Drying methods affect the extracts and essential oil of *Mentha aquatica* L. Food Biosci..

[13] Esmaeili A., Rustaiyan A., Masoudi S., Nadji K. (2006). Composition of the essential oils of *Mentha aquatica* L. and *Nepeta meyeri* Benth. from Iran. J. Essent. Oil Res..

[14] Karami H., Rasekh M., Darvishi Y., Khaledi R. (2017). Effect of drying temperature and air velocity on the essential oil content of *Mentha aquatica* L. J. Essent. Oil Bear. Plants.

[15] Zaks A., Davidovich-Rikanati R., Bar E., Inbar M., Lewinsohn E. (2008). Biosynthesis of linalyl acetate and other terpenes in lemon mint (*Mentha aquatica* var. citrata, Lamiaceae) glandular trichomes. Isr. J. Plant Sci..

[16] Chengala L., Singh N. (2017). Botanical pesticides—A major alternative to chemical pesticides: A review. Int. J. Life Sci.

[17] Pavela R., Morshedloo M.R., Mumivand H., Khorsand G.J., Karami A., Maggi F., Desneux N., Benelli G. (2020). Phenolic monoterpene-rich essential oils from Apiaceae and Lamiaceae species: Insecticidal activity and safety evaluation on non-target earthworms. Entomol. Gen..

[18] Kavallieratos N.G., Boukouvala M.C., Ntalaka C.T., Skourti A., Nika E.P., Maggi F., Spinozzi E., Mazzara E., Petrelli R., Lupidi G. (2021). Efficacy of 12 commercial essential oils as wheat protectants against stored-product beetles, and their acetylcholinesterase inhibitory activity. Entomol. Gen..

[19] Ricupero M., Biondi A., Cincotta F., Condurso C., Palmeri V., Verzera A., Zappalà L., Campolo O. (2022). Bioactivity and physico-chemistry of garlic essential oil nanoemulsion in tomato. Entomol. Gen..

[20] Desneux N., Decourtye A., Delpuech J.M. (2007). The sublethal effects of pesticides on beneficial arthropods. Annu. Rev. Entomol..

[21] Menail A.H., Boutefnouchet-Bouchema W.F., Haddad N., Taning N.T.C., Smagghe G., Loucif-Ayad W. (2020). Effects of thiamethoxam and spinosad on the survival and hypopharyngeal glands of the African honey bee (*Apis mellifera* intermissa). Entomol. Gen..

[22] Yadav I.C., Devi N.L. (2017). Pesticides classification and its impact on human and environment. Environ. Sci. Eng..

[23] Baweja P., Kumar S., Kumar G., Cham P., Kumar S., Kumar G. (2020). Fertilizers and pesticides: Their impact on soil health and environment. Soil Health.

[24] Giunti G., Benelli G., Palmeri V., Laudani F., Ricupero M., Ricciardi R., Maggi F., Lucchi A., Guedes R.N.C., Desneux N. (2022). Non-target effects of essential oil-based biopesticides for crop protection: Impact on natural enemies, pollinators, and soil invertebrates. Biol. Control.

[25] Perinelli D.R., Pavela R., Bonacucina G., Baldassarri C., Spinozzi E., Torresi J., Petrelli R., Morshedloo M.R., Maggi F., Benelli G. (2022). Development, characterization, insecticidal and sublethal effects of *Bunium persicum* and *Ziziphora* clinopodioides-based essential oil nanoemulsions on *Culex quinquefasciatus*. Ind. Crops Prod..

[26] Giordani C., Spinozzi E., Baldassarri C., Ferrati M., Cappellacci L., Santibañez Nieto D., Pavela R., Ricciardi R., Benelli G., Petrelli R. (2022). Insecticidal Activity of Four Essential Oils Extracted from Chilean Patagonian Plants as Potential Organic Pesticides. Plants.

[27] Wandjou J.G.N., Baldassarri C., Ferrati M., Maggi F., Pavela R., Tsabang N., Petrelli R., Ricciardi R., Desneux N., Benelli G. (2022). Essential Oils from Cameroonian Aromatic Plants as Effective Insecticides against Mosquitoes, Houseflies, and Moths. Plants.

[28] Benelli G., Pavela R., Giordani C., Casettari L., Curzi G., Cappellacci L., Petrelli R., Maggi F. (2018). Acute and sub-lethal toxicity of eight essential oils of commercial interest against the filariasis mosquito *Culex quinquefasciatus* and the housefly *Musca domestica*. Ind. Crops Prod..

[29] Kavallieratos N.G., Nika E.P., Skourti A., Xefteri D.N., Cianfaglione K., Perinelli D.R., Spinozzi E., Bonacucina G., Canale A., Benelli G. (2022). Piperitenone oxide-rich *Mentha longifolia* essential oil and its nanoemulsion to manage different developmental stages of insect and mite pests attacking stored wheat. Ind. Crops Prod..

[30] Van Den Dool H., Kratz P.D. (1963). A Generalization of the Retention Index System Including Linear Temperature Programmed Gas-Liquid Partition Chromatography. J. Chrom..

[31] Pavela R., Benelli G. (2016). Essential oils as ecofriendly biopesticides? Challenges and constraints. Trends Plant Sci..

[32] Taheri-Garavand A., Mumivand H., Fatahi S., Nasiri A., Omid M. (2021). Modeling the kinetics of essential oil content and main constituents of mint (*Mentha aquatica* L.) leaves during thin-layer drying process using response surface methodology. J. Food Process..

[33] Bozin B., Mimica-Dukic N., Anackov G., Zlatkovic B., Igic R. (2006). Variability of Content and Composition of *Mentha aquatica* L. (Lamiaceae) Essential Oil in Different Phenophases. J. Essent. Oil-Bear. Plants..

[34] Jerkovic I., Mastelic J. (2001). Composition of free and glycosidically bound volatiles of *Mentha aquatica* L. Croat. Chem. Acta.

[35] Malingré T.M., Maarse H. (1974). Composition of the essential oil of *Mentha aquatica*. Phytochemistry.

[36] Mimica-Dukić N., Božin B., Soković M., Mihajlović B., Matavulj M. (2003). Antimicrobial and antioxidant activities of three Mentha species essential oils. Planta Med..

[37] Atsbaha Zebelo S., Bertea C.M., Bossi S., Occhipinti A., Gnavi G., Maffei M.E. (2011). *Chrysolina herbacea* modulates terpenoid biosynthesis of *Mentha aquatica* L. PLoS ONE.

[38] Murray M.J., Hefendehl F.W. (1972). Changes in monoterpene composition of *Mentha aquatica* produced by gene substitution from *M. arvensis*. Phytochemistry.

[39] Murray M.J., Lincoln D.E. (1972). Oil composition of *Mentha aquatica*-*M. longifolia* F1 hybrids and *M. dumetorum*. Euphytica.

[40] Pavela R., Kaffkova K., Kumšta M. (2014). Chemical composition and larvicidal activity of essential oils from different *Mentha* L. and *Pulegium* species against *Culex quinquefasciatus* say (Diptera: Culicidae). Plant Prot. Sci..

[41] Pavela R. (2008). Insecticidal properties of several essential oils on the house fly (*Musca domestica* L.). Phytother. Res..

[42] Choi W.I., Lee S.G., Park H.M., Ahn Y.J. (2004). Toxicity of plant essential oils to *Tetranychus urticae* (Acari: Tetranychidae) and *Phytoseiulus persimilis* (Acari: Phytoseiidae). J. Econ. Entomol..

[43] Pandey S.K., Tandon S., Ahmad A., Singh A.K., Tripathi A.K. (2013). Structure–activity relationships of monoterpenes and acetyl derivatives against *Aedes aegypti* (Diptera: Culicidae) larvae. Pest Manag. Sci..

[44] Li A.S., Iijima A., Huang J., Li Q.X., Chen Y. (2020). Putative mode of action of the monoterpenoids linalool, methyl eugenol, estragole, and citronellal on ligand-gated ion channels. Engineering.

[45] Pajaro-Castro N., Caballero-Gallardo K., Olivero-Verbel J. (2017). Neurotoxic effects of linalool and β-pinene on *Tribolium castaneum* Herbst. Molecules.

[46] Campos E.V., Proença P.L., Oliveira J.L., Bakshi M., Abhilash P.C., Fraceto L.F. (2019). Use of botanical insecticides for sustainable agriculture: Future perspectives. Ecol. Indic..

[47] Praveena A., Sanjayan K.P. (2011). Inhibition of acetylcholinesterase in three insects of economic importance by linalool, a monoterpene phytochemical. Insect Pest Manag. Curr. Scenario.

[48] Cavanagh H.M.A., Wilkinson J.M. (2002). Biological Activities of Lavender Essential Oil. Phyther. Res..

[49] Lahlou M. (2004). Essential oils and fragrance compounds: Bioactivity and mechanisms of action. Flavour Fragr. J..

[50] Tong F., Coats J.R. (2010). Effects of monoterpenoid insecticides on [3H]-TBOB binding in house fly GABA receptor and 36Cl− uptake in American cockroach ventral nerve cord. Pestic. Biochem. Phys..

[51] Ottai M.E.S., Ahmed S.S., Din M.M.E. (2012). Genetic variability among some quantitative characters, insecticidal activity and essential oil composition of two Egyptian and French sweet basil varieties. Aust. J. Basic Appl. Sci..

[52] Hossain F., Lacroix M., Salmieri S., Vu K., Follett P.A. (2014). Basil oil fumigation increases radiation sensitivity in adult *Sitophilus oryzae* (Coleoptera: Curculionidae). J. Stored Prod. Res..

[53] Ling Chang C., Kyu Cho I., Li Q.X. (2009). Insecticidal activity of basil oil, trans-anethole, estragole, and linalool to adult fruit flies of *Ceratitis capitata*, *Bactrocera dorsalis*, and *Bactrocera cucurbitae*. J. Econ. Entomol..

[54] Vicenço C.B., Silvestre W.P., Lima T.S., Pauletti G.F. (2021). Insecticidal activity of *Cinnamomum camphora* Ness and Eberm var. *linaloolifera* Fujita leaf essential oil and linalool against *Anticarsia gemmatalis*. J. Essent. Oil Res..

[55] Vicenço C.B., Silvestre W.P., Pauletti G.F., de Barros N.M., Schwambach J. (2021). *Cinnamomum camphora* var. *linaloolifera* essential oil on pest control: Its effect on *Trialeurodes vaporariorum* (Hemiptera: Aleyrodidae). Res. Soc. Dev..

[56] Khani A., Rahdari T. (2012). Chemical composition and insecticidal activity of essential oil from Coriandrum sativum seeds against *Tribolium confusum* and *Callosobruchus maculatus*. Int. Sch. Res. Notices.

[57] Ayvaz A., Sagdic O., Karaborklu S., Ozturk I. (2010). Insecticidal activity of the essential oils from different plants against three stored-product insects. J. Insect Sci..

[58] Cheng J., Yang K., Zhao N.N., Wang X.G., Wang S.Y., Liu Z.L. (2012). Composition and insecticidal activity of the essential oil of *Cananga odorata* leaves against *Sitophilus zeamais* Motschulsky (Coleoptera: Curculionidae). J. Med. Plant Res..

[59] Pavela R., Ferrati M., Spinozzi E., Maggi F., Petrelli R., Rakotosaona R., Ricciardi R., Benelli G. (2022). The Essential Oil from the Resurrection Plant *Myrothamnus moschatus* Is Effective against Arthropods of Agricultural and Medical Interest. Pharmaceuticals.

[60] WHO (1996). Report of the WHO Informal Consultation on the Evaluation and Testing of Insecticides.

[61] Abbott W.S. (1925). A method of computing the effectiveness of an insecticide. J. Econ. Entomol..

[62] Finney D.J. (1971). Probit Analysis.

